# Imaging of human cells exposed to an antifungal antibiotic amphotericin B reveals the mechanisms associated with the drug toxicity and cell defence

**DOI:** 10.1038/s41598-018-32301-9

**Published:** 2018-09-14

**Authors:** Ewa Grela, Mateusz Piet, Rafal Luchowski, Wojciech Grudzinski, Roman Paduch, Wieslaw I. Gruszecki

**Affiliations:** 1 Department of Biophysics, Institute of Physics, Maria Curie-Skłodowska University, Lublin, Poland; 2 Department of Biophysics, Institute of Biology and Biochemistry, Maria Curie-Skłodowska University, Lublin, Poland; 3 Department of Virology and Immunology, Institute of Microbiology and Biotechnology, Maria Curie-Skłodowska University, Lublin, Poland; 4 Department of General Ophthalmology, Medical University of Lublin, Lublin, Poland

## Abstract

Amphotericin B is an antibiotic used in pharmacotherapy of life-threatening mycotic infections. Unfortunately, the applicability of this antibiotic is associated with highly toxic side effects. In order to understand molecular mechanisms underlying toxicity of amphotericin B to patients, two cell lines, human normal colon epithelial cells (CCD 841 CoTr) and human colon adenocarcinoma cells (HT-29) were cultured in the presence of the drug and imaged with the application of fluorescence lifetime imaging microscopy and Raman scattering microscopy. The results of the cell viability assays confirm high toxicity of amphotericin B towards human cells. The images recorded demonstrate effective binding of amphotericin B to biomembranes. Analysis of the images reveals the operation of a defence mechanism based upon the elimination of molecules of the drug from living cells via formation of small amphotericin B-containing lipid vesicles. The fact that exosomes formed are devoid of cholesterol, as concluded on the basis of the results of Raman analysis, suggests that sequestration of sterols from the lipid phase of biomembranes is not a sole mechanism responsible for the toxic side effects of amphotericin B. Alternatively, the results imply that molecules of the drug present directly within the hydrophobic membrane core disturb the lipid membrane structure and affect their biological functions.

## Introduction

The dramatic increase in mycotic infections, we are facing nowadays, is a challenge for researchers working on developing effective antifungal drugs^1,2^. Amphotericin B (AmB) has been used as a gold standard to treat life-threatening, systemic mycoses, despite high toxicity of this drug to patients, owing to the high performance of this antibiotic^3^ (see Supplementary Information Fig. S1 for a chemical structure). According to a current knowledge, biomembranes of human and fungi cells are a primary target of the drug and both the therapeutic and toxic side effects of AmB are based upon impairing of physiological processes taking place in membranes. Among the possible mechanisms of action of the drug towards biomembranes, a disruption of the physiological ion transport via the pore-like, transmembrane structures of AmB, are considered to be highly effective and probable^4–7^. Alternatively, an effect of AmB on structural properties of biomembranes via destabilization of their lipid phase and resulting impairment of the membrane functionality was postulated^8–10^. Recently, another molecular mechanism has been proposed, which consists in impairing of biomembranes functionality via sequestration of sterols from the lipid phase of biomembranes, owing to the formation of extramembraneous AmB-sterol structures^11^. The results of our recent research based on fluorescence lifetime imaging microscopy (FLIM) of single model lipid membranes has confirmed the possibility of formation of such structures^12,13^. On the other hand, the results of the present study carried out on human living cell cultures exposed to AmB seem to suggest the operation of different mechanisms in natural systems. Owing to the toxicity of AmB to patients, intravenous infusions of different formulations of the drug are nowadays a recommended delivery mode, in order to bypass a digestive tract. We selected human normal colon epithelial cells (CCD 841 CoTr) for the purpose of the present work aimed to investigate the molecular mechanisms of toxicity of AmB to cells and protection against them. In order to understand molecular mechanisms underlying potential toxicity of AmB, also to oncological patients of selected alimentary tract tumour, additionally, human colon adenocarcinoma cells (HT-29) were cultured in the presence of the drug and imaged with the application of fluorescence lifetime imaging microscopy and Raman scattering microscopy.

## Results

**Figure 1:**
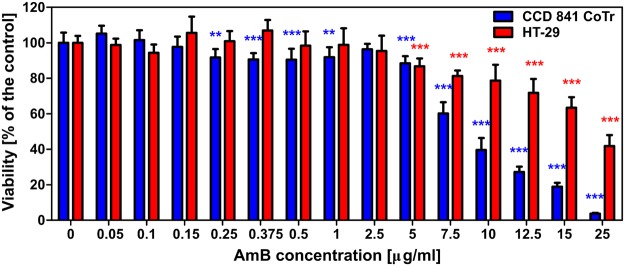
Results of viability assays of cells cultured under presence of AmB. AmB concentration profiles of cell viability from the cell lines CCD 841 CoTr and HT-29, determined according to the Neutral Red method (one-way Anova, post-hoc: Dunnett test; *p < 0.05, **p < 0.01; ***p < 0.005).

**Figure 2:**
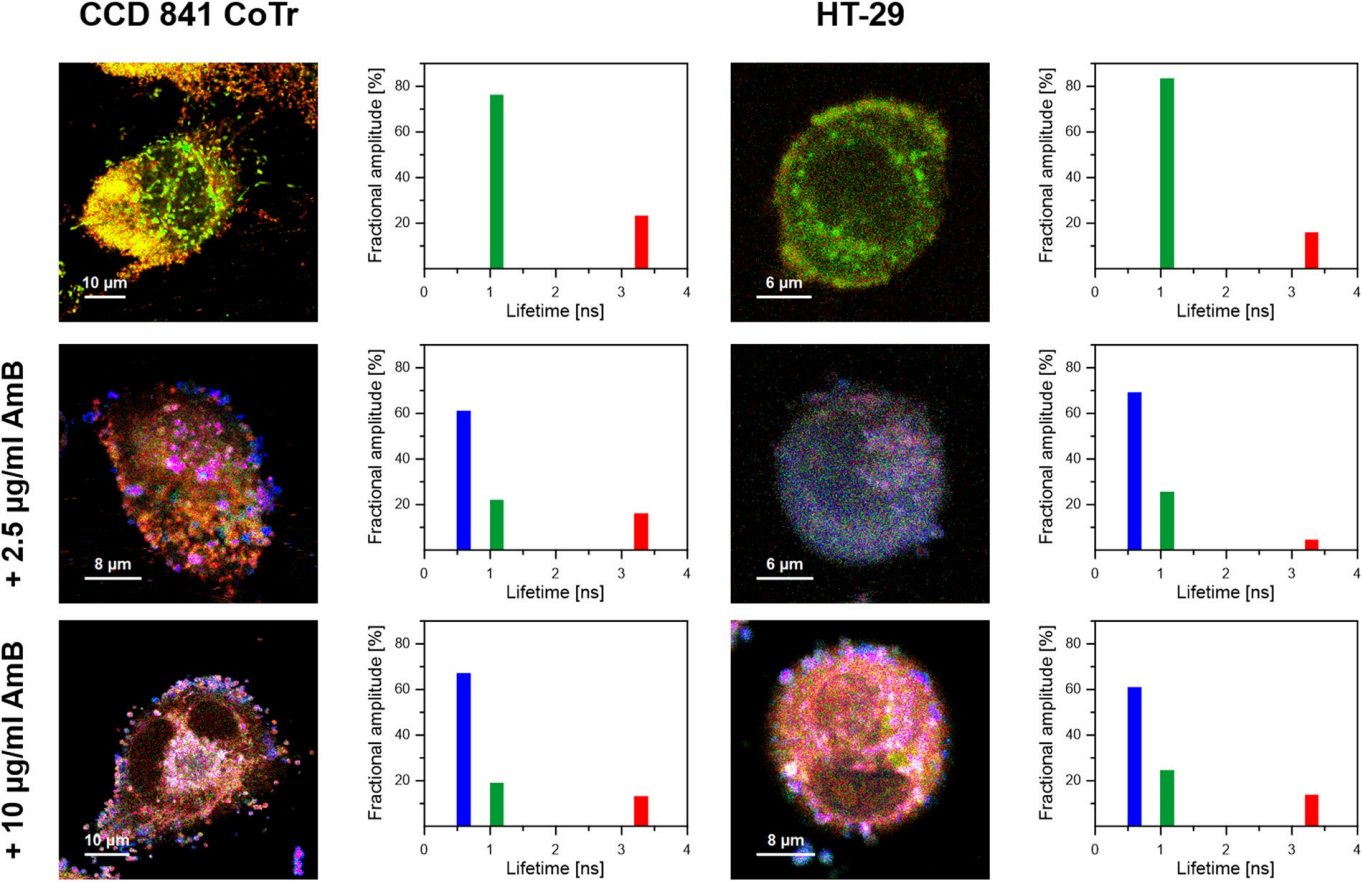
Fluorescence lifetime images of cells. Fluorescence lifetime imaging data of two human cell lines: CCD 841 CoTr (on the left) and HT-29 (on the right), cultured without additions or with the addition of AmB at a concentration of 2.5 µg/ml and 10 µg/ml (marked). Cells were cultured on glass coverslips and imaged on the same slides (*in situ*). Side panels present the results of detailed fluorescence lifetime analysis in the images presented. A height of bars represents a fractional contribution of each lifetime component. The colour codes correspond to the localization of lifetime components on images: 1.1 ns green, 3.3 ns red and 0.6 ns blue.

**Figure 3:**
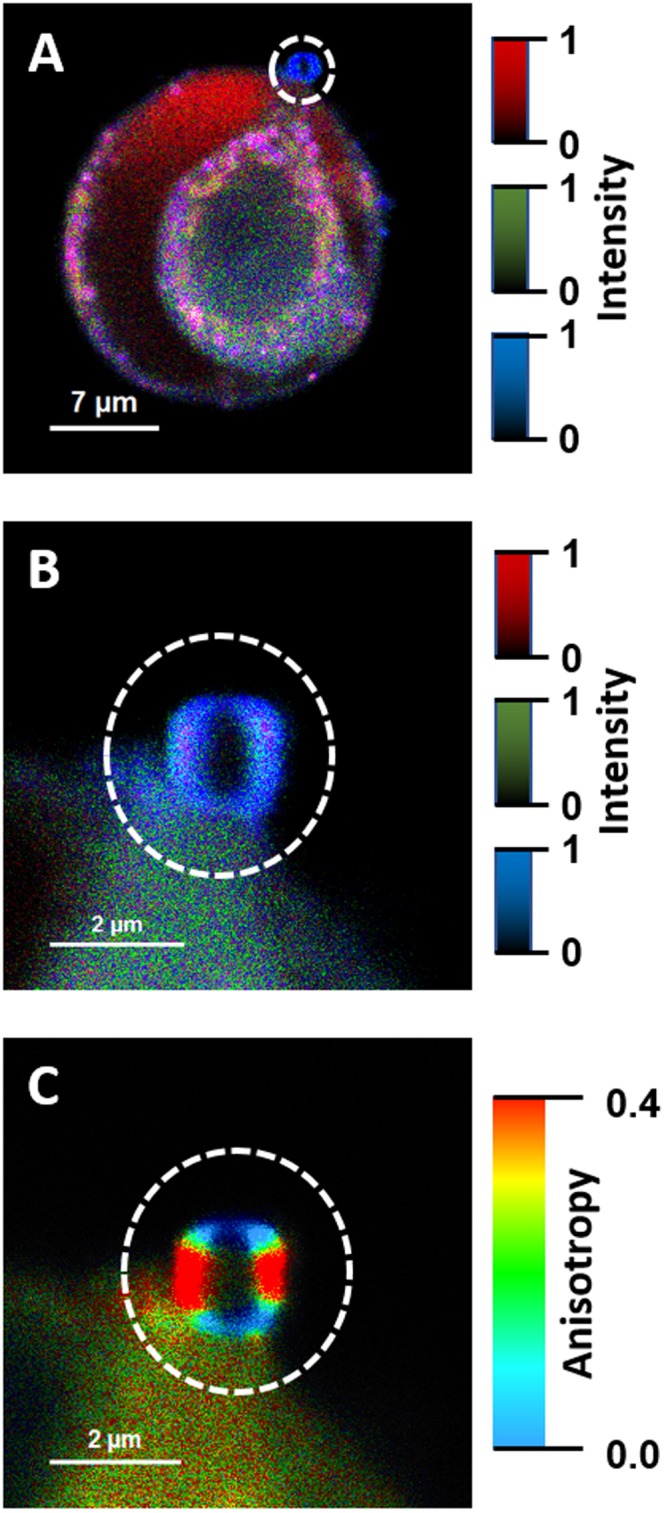
Fluorescence lifetime and fluorescence anisotropy images of a single exosome. Panel A shows a cell from the HT-29 cell line, cultured at AmB concentration of 10 µg/ml. Panel B shows an image of a single exosome vesicle, selected in panel A (marked with a white circle) and recorded with higher resolution. Panel C shows the same exosome imaged based on fluorescence anisotropy data. The colour code for panels A and B as in Fig. 2. The relatively short fluorescence lifetimes of AmB in the exosome (panel B) is indicative of the formation of small aggregated forms of the drug in the lipid phase. The relatively high fluorescence anisotropy values at the left- and right-hand of the image of a vesicle cross-section (panel C) are consistent with the perpendicular orientation of AmB molecules with respect to the membrane plane.

**Figure 4:**
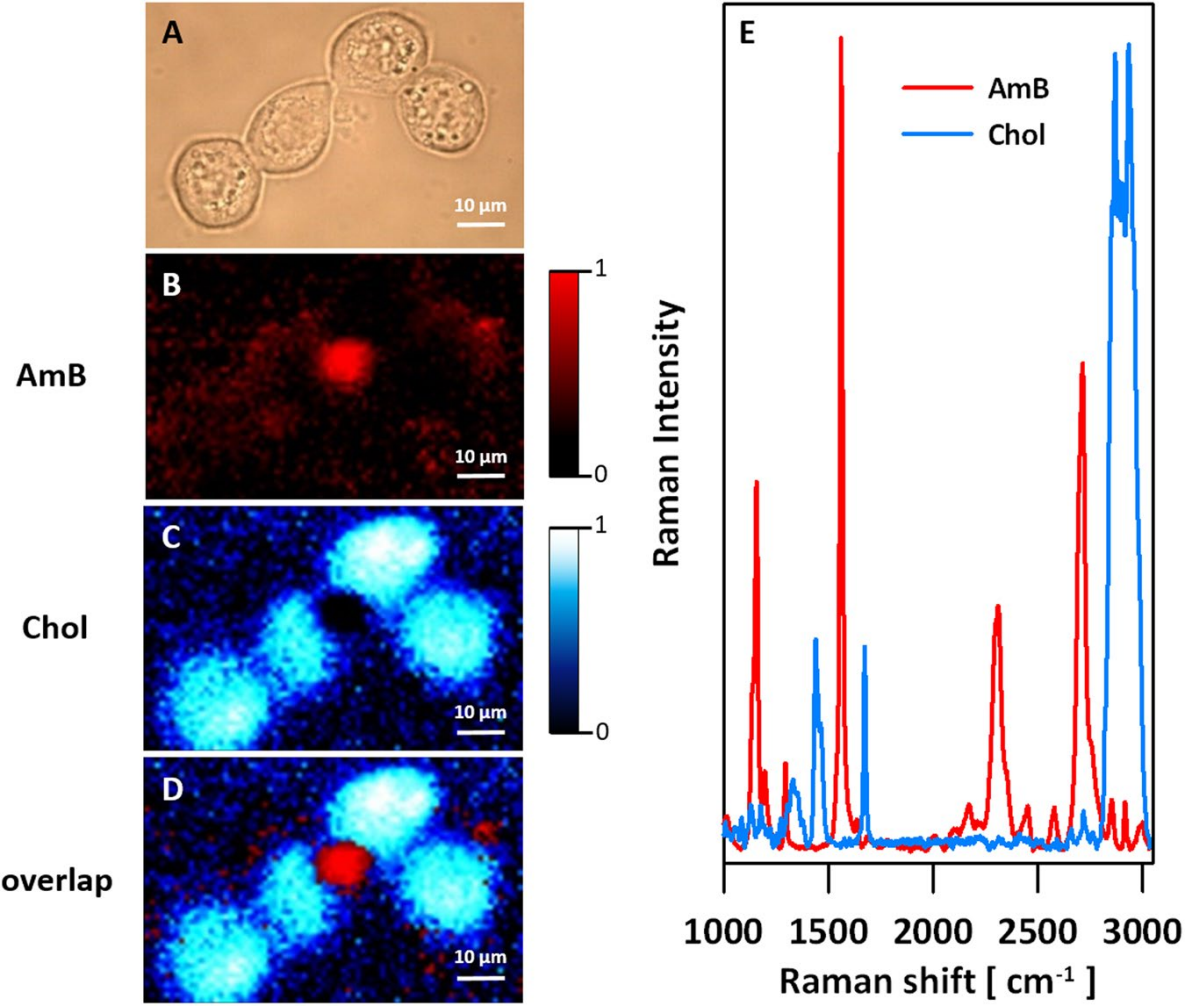
Images of an HT-29 single cell from the culture grown in the presence of AmB. The concentration of AmB in the growing medium was 5 μg/ml. (**A**) Optical image. (**B**–**D**) Raman images: B – distribution of AmB, C – distribution of Chol, D – overlap of images presented in panels B and C. Panel E shows the template Raman spectra recorded for pure AmB and Chol under the same conditions and used for analysis of localization of the antibiotic and the sterol in the cells imaged.

Two human cell lines, CCD 841 CoTr and HT-29, were cultured in the presence of AmB in a concentration range of 0.05 to 25 μg/ml in the growth medium. As expected, higher concentrations of the antibiotic are toxic to human cells (above 5 μg/ml, see Fig. 1). Both CCD 841 CoTr and HT-29 cells were susceptible to AmB, but up to a concentration of 5 μg/ml the cytotoxic effect did not exceed 15% compared to the control (viability inhibition to 88.4 and 86.8% in CCD 841 CoTr and HT-29 cell cultures, respectively). At concentrations higher than 5 μg/ml the drastic fall of cells viability was noted and the effect towards normal cells (viability decreased to 3.6% compared to the control) was more severe than on cancer cells (inhibition to 41.8% of the control). IC50 values were 8.7 μg/ml for CCD 841 CoTr and 21.2 μg/ml for HT-29 cells. Cells cultures were imaged with the application of FLIM and Raman scattering microscopy. Figure 2 presents FLIM images of CCD 841 CoTr and HT-29 cells, control and cultured in the presence of AmB (2.5 and 10 μg/ml). As can be seen, the cells from both the cell lines demonstrate auto-fluorescence characterized by two fluorescence lifetime components: 1.1 ns and 3.3 ns, very close to the fluorescence lifetime components reported previously^14^. Exposition of cells to AmB present in the growth medium results in binding of the drug molecules to cells, manifested by the appearance of additional fluorescence lifetime component τ = 0.6 ns. A fluorescence lifetime level of this component (below 1 ns) suggests that AmB in the cells imaged appears in the form of small supramolecular structures^12,13,15^. Interestingly, apart of AmB distributed homogenously in the cells imaged and represented by the pixels coded with blue colour, the numerous nanoscale structures can be resolved, visible particularly at higher concentrations of the drug (Fig. 2). Such distinct structures, selected on the large-scale images, additionally were imaged with higher resolution. Examples of such images are presented in Figs 3 and S2. The AmB-containing nanostructures observed in the images can be concluded to be created in a process of a secretion by budding cell membrane fragments. Several subsequent phases of this process can be distinguished in the images. Figure 3 presents the images of a mature exosome being still attached to the cell membrane. A distinctly blue colour of the FLIM image of the exosome, representing short fluorescence lifetime component assigned to AmB, with a minimum contribution from a green colour-coded and red colour-coded autofluorescence of the cell, is a manifestation of different composition of the plasma membrane and the newly formed lipid vesicle. The fact that the fluorescence intensity in the cross-section of the AmB-containing vesicle is more intensive in the left-hand and right-hand parts than in the upper and lower parts is a clear demonstration that fluorophores of the antibiotic are oriented vertically with respect to the membrane plane^12,13,16^. Such a conclusion is based on the photoselection and has very strong support from the distribution of fluorescence anisotropy values in the structure imaged in Fig. 3 (see Fig. S3 for interpretation of fluorescence anisotropy values). This means that molecules of AmB are loaded into the hydrophobic core of the lipid vesicles and that they are oriented perpendicular with respect to the membrane plane. Virtually the same fluorescence lifetime values of AmB were recorded in the cells and in the exosomes which implies the same molecular organization of molecules of the antibiotic before and after an exosome formation. A level of AmB fluorescence lifetime (below 1 ns) and fluorescence emission spectra recorded based on a micro-spectroscopy approach (Fig. S4), confirm that molecules of the antibiotic accommodated into the membranes and localized in the exosomes are associated in small supramolecular structures. In order to check whether the AmB-carrying exosomes are also rich with cholesterol (Chol) extracted from cell membranes, the samples were imaged with the application of Raman microscopy. Figure 4 presents the results of Raman imaging of cells cultured under exposure to AmB present in the growing medium. The collections of the Raman spectra recorded during cell imaging were deconvoluted based on the template spectra of pure AmB and Chol. This enabled to selectively image a distribution of AmB (Fig. 4B) and Chol (Fig. 4C). Interestingly, the Raman imaging results reveal that AmB-containing exosomes are devoid of Chol. The same phenomenon could be observed both in the case of HT-29 cells (Fig. 4) and CCD 841 CoTr cells (Fig. S5).

## Discussion

**Figure 5:**
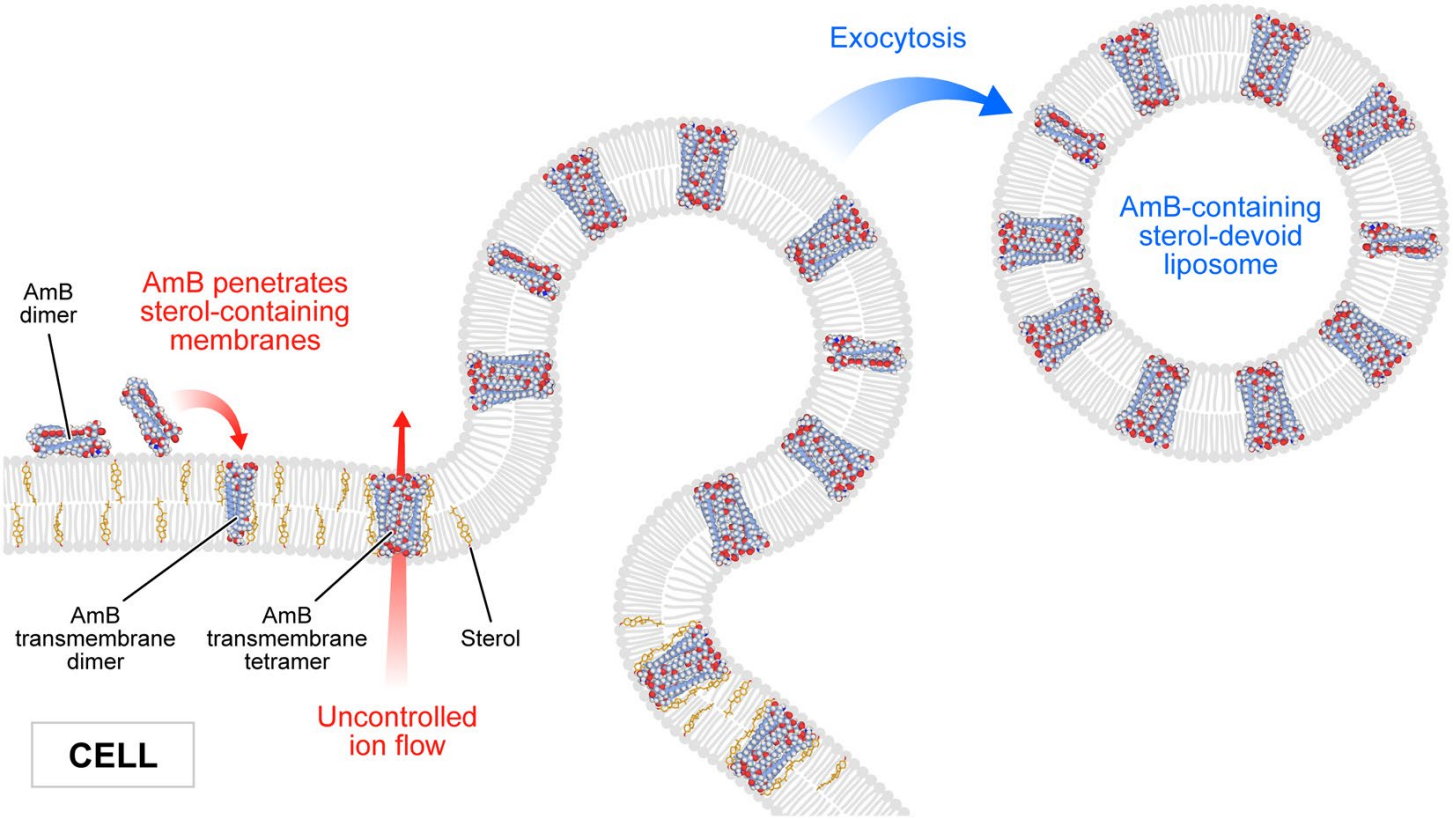
Schematic representation of binding and extrusion of amphotericin B to/from biomembranes. The model is discussed in the text.

In the present work, we analysed binding of molecules of antifungal antibiotic AmB to human cells from two cell lines, normal colon epithelial cells and colon adenocarcinoma cells, by means of imaging with fluorescence lifetime and Raman scattering microscopies. Assays of the viability of cells exposed to AmB, run prior to imaging, confirmed high toxicity of this antifungal antibiotic also for human cells. Interestingly, the resistance of normal cells to the toxicity of the drug has been determined significantly lower as compared to adenocarcinoma cells (Fig. 1). One of the possible explanations of such a difference may be associated with a different membrane protein composition in normal and cancer cells^17^. It may influence not only on the pathogenicity of tumour cells but also distinct sensitivity to cytotoxic or antifungal drugs. Another explanation could be based upon high proliferation rate of cancer cells, involving remodelling of plasma membrane systems and therefore facilitated exocytosis resulting in the elimination of AmB molecules from the cell membranes (see Fig. 3). Such a process has been observed in all the AmB-containing cells imaged in the present study and therefore it can be assumed to be a natural defence mechanism against toxication with this antibiotic. The quantitative analysis of amplitudes of the short-lifetime fluorescence components representing AmB bound to the cells shows that independently of the actual concentration of the antibiotic in the growing medium an amplitude does not exceed ~60% (4-fold increase in the AmB concentration, see Fig. 2). This seems to be a demonstration of an efficient operation of a certain defence mechanism, for example, based upon the elimination of AmB molecules from the cells. On the other hand, it may not be excluded that this is a maximum concentration of the drug (corresponding to 60% amplitude of the short lifetime fluorescence component) which can be resisted by a living cell, before cell death, providing that only alive cells were subjected to imaging. Interestingly, the exosomes formed, loaded with AmB, have been found not to contain Chol (see Figs 4 and S5). Such a result is very far from our expectations, taking into consideration the fact that AmB can effectively bind from the water phase to lipid membranes exclusively those containing sterols in their hydrophobic core^12,13,18^. The results of the present work show that indeed, AmB binds to Chol-containing membranes but, on the other hand, can diffuse within the lipid phase and can be effectively eliminated via exocytosis from the membrane domains characterized by a relatively low level of sterols. A scheme summarizing those findings is presented in Fig. 5. The fact that despite the operation of such a defence mechanism, AmB is highly toxic to human cells, shows that molecules of the drug present in the plasma membranes affect the membrane physiological functionality. According to the results of the fluorescence lifetime and fluorescence spectral analyses, AmB incorporated into the cells appears in the form of small supramolecular structures. It is very likely that such structures promote uncontrolled ion flow through biomembranes, affecting electrostasis of living cells. The results of the present study show that not necessary sequestration of sterols from the lipid phase but also the direct presence of molecules of the drug within plasma membranes can affect their biological functions and is, at least partially, responsible for the toxicity of AmB. We also report operation of the detoxification mechanism based upon the elimination of AmB from the cell membranes via extrusion of liposomes loaded with the antibiotic.

## Methods

### Chemicals and preparation

Antibiotic amphotericin B from *Streptomyces* sp., dimethyl sulfoxide (DMSO) and cholesterol were purchased from Sigma Aldrich (USA). All other chemicals used in the preparations were of analytical grade. Water was purified by a Milli-Q system from Merck Millipore (France).

In order to additionally purify AmB, a powder of the antibiotic was suspended in a mixture of water and chloroform (1:1, v/v) and vortexed for 30 min, then collected from the interphase (this procedure has been repeated three times)^15,19^. Finally, AmB was evaporated under gaseous nitrogen.

A concentration of the antibiotic was determined based on a molar extinction coefficient of AmB in DMSO solution (121 400 M^−1^ cm^−1^) at 416 nm absorption maximum^20^. For this purpose, electronic absorption spectra were recorded with the application of a Cary 60 UV-Vis spectrophotometer from Agilent Technologies (Australia).

### Cell culture

The research was held on two cell lines: human normal colon epithelial cells CCD 841 CoTr (ATCC No. CRL-1807) and human adenocarcinoma cells HT-29 (ATCC No. HTB-38) derived from a stage I cancer. Cells were cultured in 5% CO_2_/95% air humidified atmosphere in a mixture of DMEM: RPMI 1640 (1:1) media (Sigma, St Louis, MO, USA) at 34 °C (CCD 841 CoTr) and RPMI 1640 medium at 37 °C (HT-29). Media were supplemented with 2% or 10% (v/v) FBS (Gibco^TM^, Paisley, UK) and antibiotics: 100 U/ml penicillin, 100 μg/ml streptomycin (Sigma).

For the experiments, cells were treated with 25 mM trypsin and EDTA, counted and diluted to a density of 1 × 10^5^ cells/ml. For neutral red (NR) assay, 100 μl of cell suspension were poured to each well of 96-well plate, and for FLIM and Raman analyses, 2 ml of cells were poured onto round glass cover slips placed inside the Petri dish. Subsequently, cultures were incubated for 24 h with appropriate concentrations of AmB. Afterwards, for the FLIM and Raman analyses, cells were washed twice with PBS.

### Viability assay by Neutral Red uptake

The method is based on uptake of supravital dye, neutral red, by live cells and its accumulation in the lysosomes. The absorbance is directly proportional to the viability of the cells^21,22^. After 24 h incubation with AmB at concentrations of range from 0.05 to 25 μg/ml, the medium was discarded and 100 μl of NR dye (40 μg/ml)(Sigma) were added to each well. Plates were incubated for 3 h in a humidified atmosphere at 34 or 37 °C. Afterwards, the dye-containing medium was removed and cells were fixed with 200 μl of 0.5% formalin in 1% CaCl_2_. Subsequently, the fixative was removed and dye was solubilized with 100 μl of solvent (1% acetic acid in 50% ethanol). The plates were gently shaken for 20 min at room temperature and the absorbance of the extracted dye was measured spectrophotometrically at 540 nm using a microplate reader (Molecular Devices Corp., Emax, Menlo Park, CA).

Results of viability are presented as arithmetic mean ± SD and were analyzed using GraphPad Prism software. Statistical significance was calculated with one-way ANOVA test and Dunnett post-hoc test and is expressed with *, where * signifies p < 0.05, **p < 0.01, and ***p < 0.001. According to FDA, the maximum recommended a total daily dose of amphotericin B desoxycholate for adults should not exceed 1.5 mg/kg.

### Fluorescence Lifetime Imaging Microscopy and micro-spectroscopy

Fluorescence lifetime imaging was carried out on MicroTime 200 (Picoquant GmbH, Germany) linked with Olympus IX71 inverted microscope. The cell samples were illuminated by 405 nm pulsed laser with 10 MHz repetition frequency and 16 ps resolution time. During the experiments was used a silicon oil-immersed objective (NA = 1.3, 60×). Measurements were carried out with the application of ZT 405RDC dichroic, ZET405 StopLine Notch Filter, 430 long wavelength-pass filters from Chroma-AHF Analysentechnik and a confocal pinhole of 50 μm in diameter. The fluorescence signal was divided into perpendicular- and parallel-polarized channels and was simultaneously measured by two twin Single Photon Avalanche Diodes. The perpendicular (F_⊥_) and parallel (F_‖‖_) intensities were further used to calculate the anisotropy as defined:

$$r=\frac{{F}_{\Vert }-G{F}_{\perp }}{{F}_{\Vert }+2G{F}_{\perp }}$$

Polarization directions are referred to the polarization of excitation laser beam. G was an instrumental correction factor (typically 1.01). A value of factor G was determined before each experiment, in separate measurement carried out with a long-lifetime fluorescence probe.

Fluorescence lifetimes and fluorescence anisotropy values were analyzed and determined with the application of SymPhoTime 64 v. 2.3 software (Picoquant GmbH, Germany).

Fluorescence emission spectra of micro-scale objects were recorded from a selected area of the imaged cells with the application of a Shamrock 163 spectrograph coupled with the microscope. For detection, a single photon-sensitive camera (Newton EMCCD DU970P BUF, Andor Technology) was used, cooled to minus 50 °C.

### Raman imaging

Raman spectral analysis and imaging on a microscale was carried out with inVia Reflex confocal Raman microscope (Renishaw, UK) with Cobolt o8-NLD 405 nm laser (power at a sample 0.2 mW). Water immersed objective (Olympus NA = 1.2, 60×) was applied during the experiments. In each pixel of the map, a Raman spectrum was recorded in the region between 953–3033 cm^−1^, with the application of a 2400 lines/mm grating (1 cm^−1^ spectral resolution) and EMCCD Newton 970 camera (Andor Technology, UK) cooled to minus 50 °C. Spectrum acquisition time was set to 0.1 s. All results were analyzed by DCLS spectral deconvolution using Wire 4.4 software (Renishaw, UK).

## Electronic supplementary material

Supplementary Information
