# Supplementary material for: Imaging of human cells exposed to an antifungal antibiotic amphotericin B reveals the mechanisms associated with the drug toxicity and cell defence
Source: Sci Rep. 2018 Sep 14;8:14067. doi: 10.1038/s41598-018-32301-9 (PMC6138690; doi:10.1038/s41598-018-32301-9)
Supplement: Supplementary file 1 — Supplementary Information [file 41598_2018_32301_MOESM1_ESM.pdf]

Supplementary Information

Imaging of human cells exposed to an antifungal antibiotic amphotericin B reveals the mechanisms associated with the drug toxicity and cell defence

Ewa Grela[1,2], Mateusz Piet[3], Rafal Luchowski[1], Wojciech Grudzinski[1], Roman Paduch[3,4] & Wieslaw I. Gruszecki[1,]*

1. Department of Biophysics, Institute of Physics, Maria Curie-Skłodowska University, Lublin, Poland
2. Department of Biophysics, Institute of Biology, Maria Curie-Skłodowska University, Lublin, Poland
3. Department of Virology and Immunology, Institute of Microbiology and Biotechnology, Maria Curie-Skłodowska University, Lublin, Poland
4. Department of General Ophthalmology, Medical University of Lublin, Lublin, Poland.

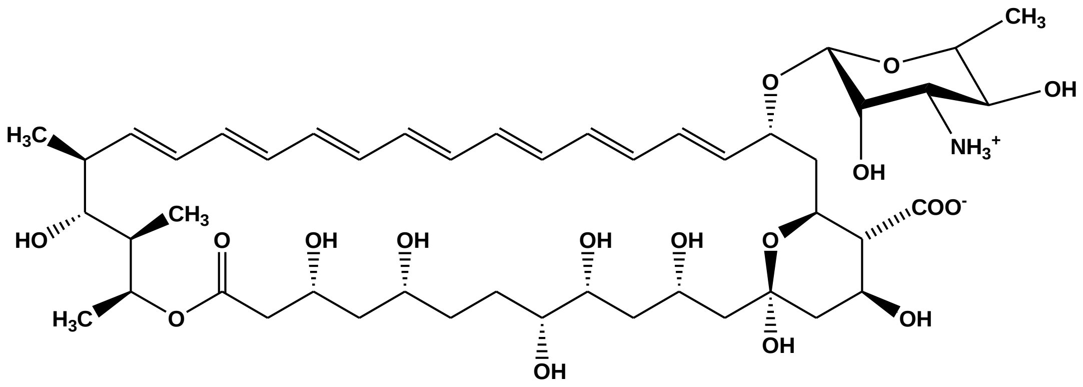

Figure S1 Chemical structure of amphotericin B molecule.

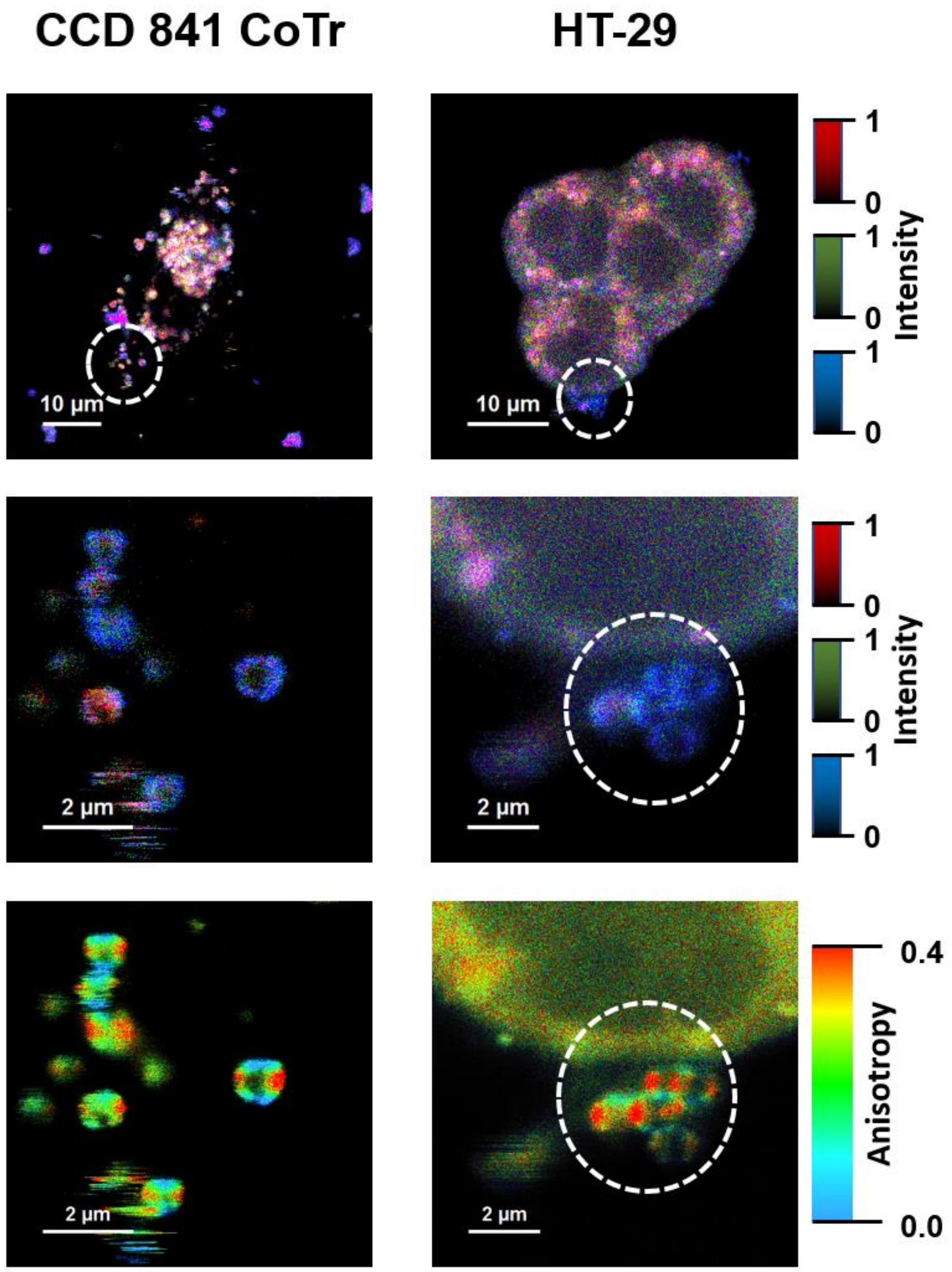

Figure S2 FLIM and fluorescence images of cells cultured under the presence of antibiotic AmB. The exosomes selected in the large-scale images (upper panels) are imaged with higher resolution by means of FLIM (middle panels) or fluorescence anisotropy (lower panels).

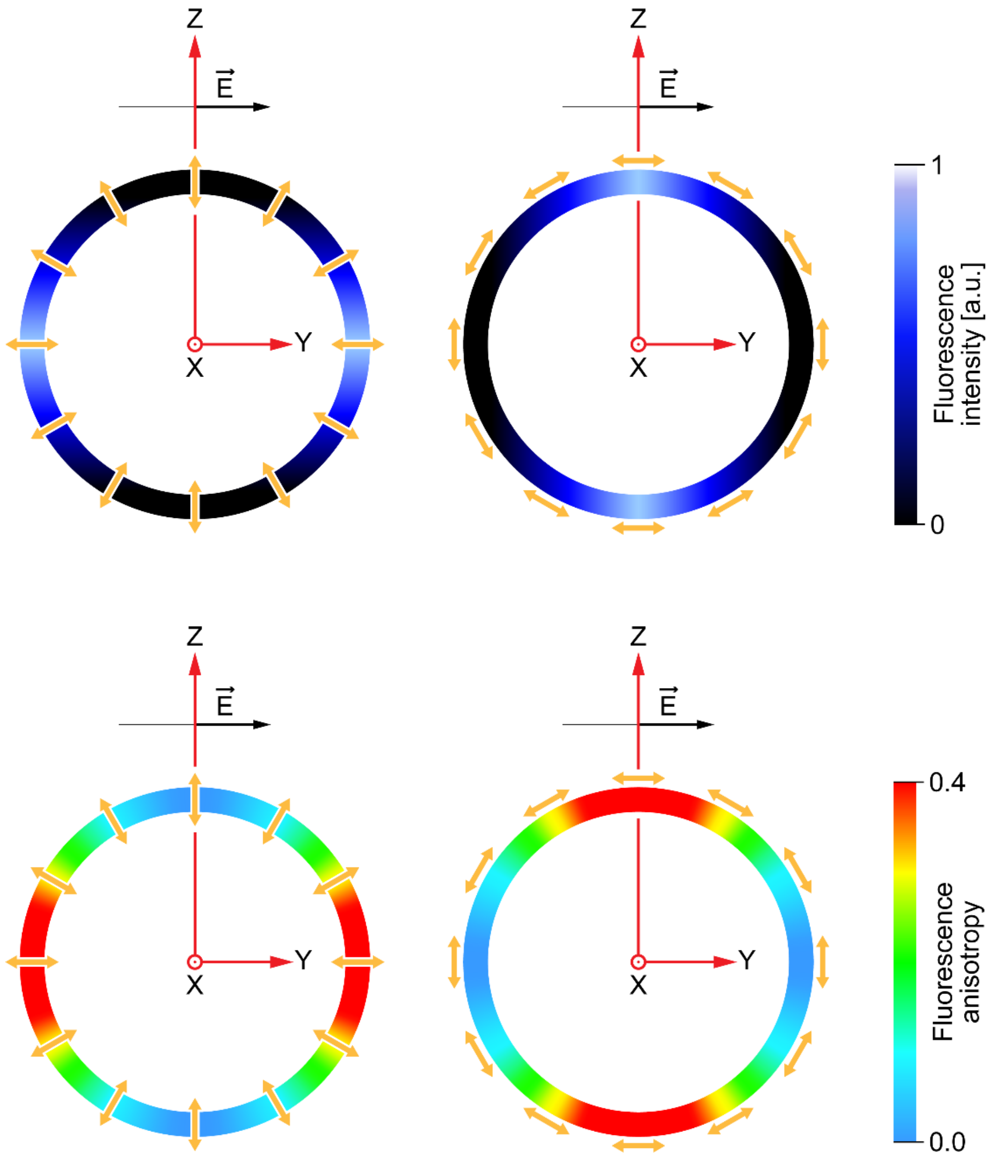

Figure S3 Schematic representation of the idea of photoselection. Linear fluorophores represented by yellow arrows are bound to the membranes and oriented perpendicular (on the left) or parallel (on the right) with respect to the membrane plane of a lipid vesicle. Images represent an equatorial cross-section of a lipid vesicle imaged by means of fluorescence (upper panels) or fluorescence anisotropy (lower panels).

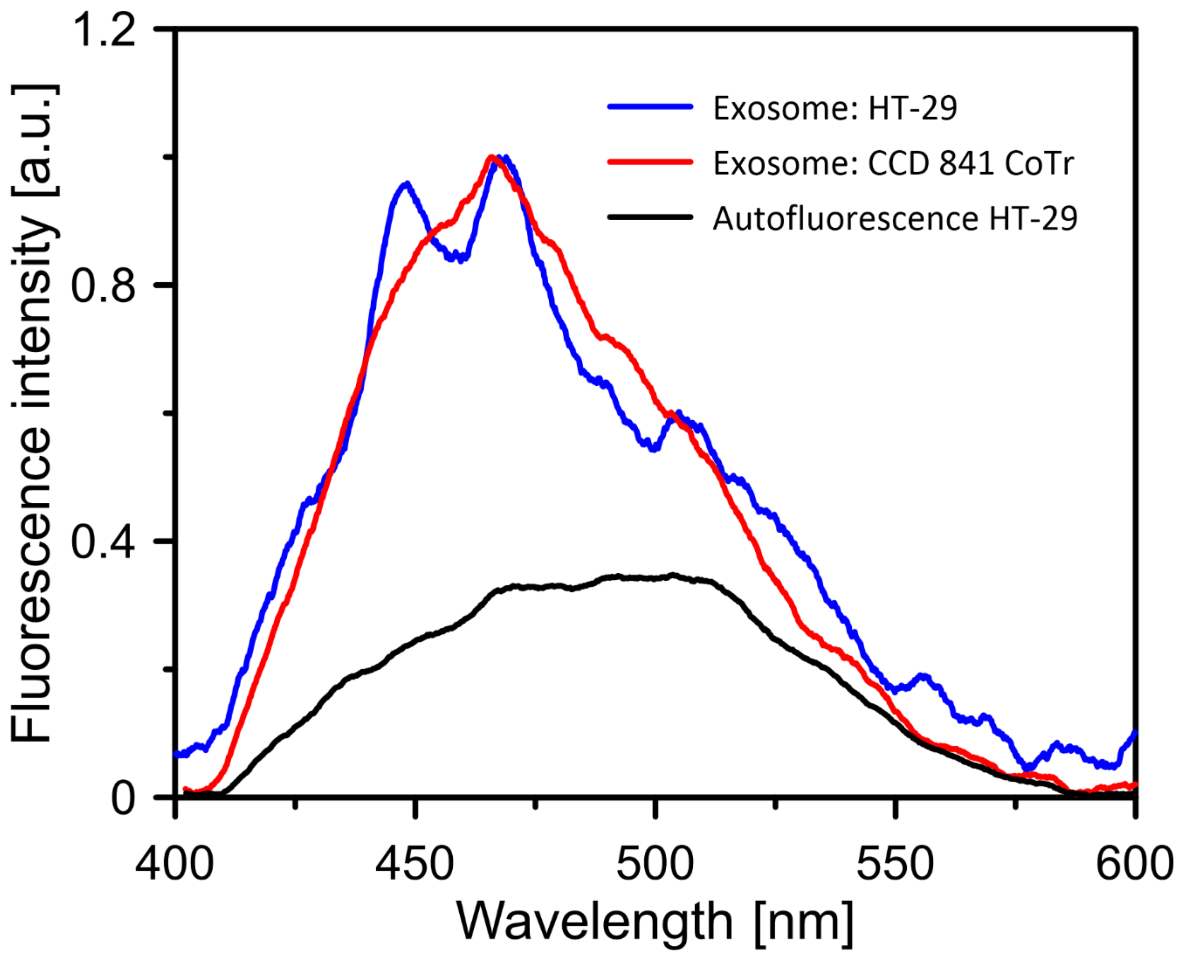

Figure S4. Fluorescence emission spectra recorded from selected nanoscale areas of microscopic images of cells. The spectra recorded from the exosomes of CCD 841 CoTr and HT-29 cells cultured under the presence of AmB in a growing medium (10 μg/ml) and from a control HT-29 cell (autofluorescence). The spectra recorded from the AmB-containing membranes were normalized at the maximum (~470 nm) while the autofluorescence spectrum was scaled in order not to exceed the spectra of the samples comprising additional fluorescing component.

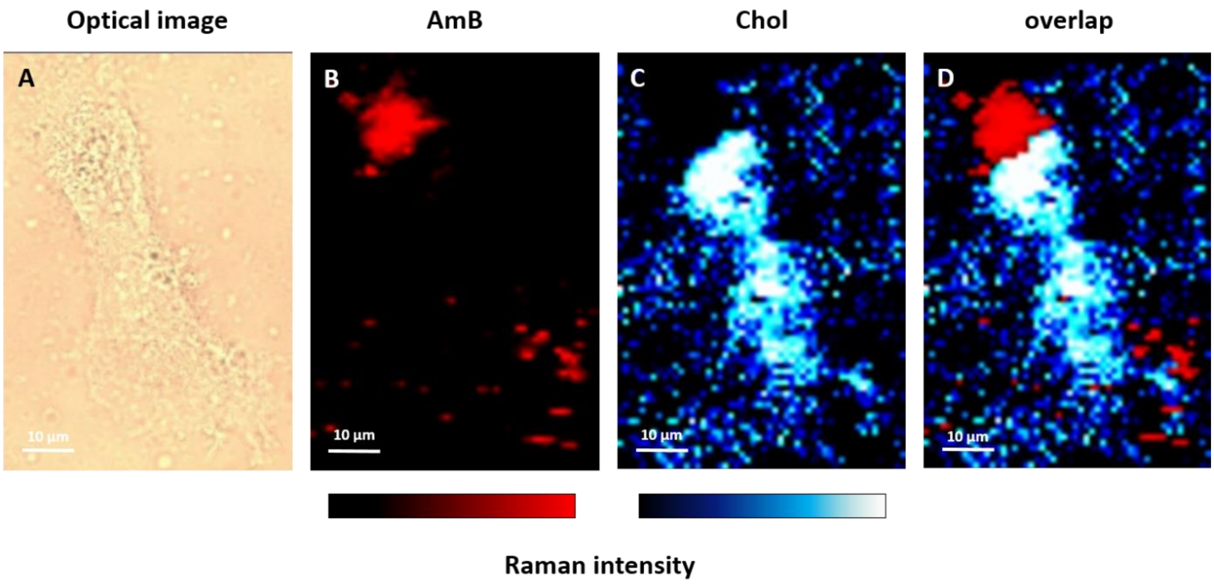

Figure S5. Images of a CCD 841 CoTr single cell from the culture grown in the presence of AmB. The concentration of AmB in the growing medium was 5 µg/ml. (A) Optical image, (B-D) Raman images: B – distribution of AmB, C – distribution of Chol, D – overlap of images presented in panels B and C. The analysis procedure the same as in the case of the images presented in Figure 4 of the paper.
